# Bibliometric analysis of scientific articles published in Brazilian and international orthodontic journals over a 10-year period

**DOI:** 10.1590/2176-9451.19.2.056-065.oar

**Published:** 2014

**Authors:** Neudí Antonio Primo, Vivian Bertoglio Gazzola, Bruno Tochetto Primo, Maximiano Ferreira Tovo, Italo Medeiros Faraco Junior

**Affiliations:** 1 Professor, Specialization course in Orthodontics, FUNORTE; 2 Doctorate student in Orthodontics, Lutheran University of Brazil (ULBRA); 3 MSc in Oral and Maxillofacial Surgery and Traumatology, ULBRA; 4 Professor, Doctorate and Master's program in Dentistry, ULBRA

**Keywords:** Bibliometrics, Dental research, Orthodontics, Study characteristics

## Abstract

**Objective:**

This study aimed at describing the profiles of Brazilian and international studies published in orthodontic journals.

**Methods:**

The sample comprised 635 articles selected from two scientific journals, i.e., Dental Press Journal of Orthodontics and American Journal of Orthodontics and Dentofacial Orthopedics, which were analyzed at three different intervals over a 10-year period (1999 - 2004 - 2009). Articles were described in terms of knowledge domain, study design, and country of origin (or state of origin for Brazilian papers).

**Results:**

The most frequent study designs adopted in international studies were cohort (23.9%) and cross-sectional (21.7%) designs. Among Brazilian papers, cross-sectional studies (28.9%) and literature reviews (24.6%) showed greater frequency. The topics most often investigated were dental materials (17%) and treatment devices (12.4%) in international articles, with the latter topic being addressed by 16% of the Brazilian publications, followed by malocclusion, with 12.6%. In all cases, the most frequent countries of origin coincided with the countries of origin of each journal.

**Conclusions:**

The majority of the studies analyzed featured a low level of scientific evidence. Moreover, the findings showed that journals tend to publish studies produced in their own country of origin, and that there are marked discrepancies in the number of papers published by different Brazilian states.

## Abstract (pt)

**Objetivo:**

determinar o perfil das publicações nacionais e internacionais nos periódicos de Ortodontia.

**Métodos:**

foram avaliados 635 artigos da Revista Dental Press de Ortodontia e Ortopedia Facial e American Journal of Orthodontics and Dentofacial Orthopedics, em três tempos distintos, dentro de um intervalo de 10 anos (1999, 2004 e 2009), caracterizando as áreas de conhecimento, os delineamentos utilizados e identificando países e estados brasileiros com maior produção científica.

**Resultados:**

os delineamentos dos estudos internacionais mais utilizados foram estudos de coorte (23,9%) e transversal (21,7%). Em estudos nacionais, destacam-se os delineamentos transversais (28,9%) e revisão de literatura (24,6%). Nas publicações internacionais, as categorias temáticas mais pesquisadas foram materiais dentários (17%), além de dispositivos de tratamento (12,4%); essa mesma categoria esteve presente em 16% das publicações nacionais, e a má oclusão dentária em 12,6%. A nacionalidade das publicações mais observada foi a do próprio país de origem do periódico.

**Conclusões:**

a maioria dos artigos publicados refere-se a estudos de menor potencial para estabelecer evidências científicas. Além disso, a situação detectada ilustra a propensão das revistas publicarem os artigos produzidos dentro de seu país de origem, bem como as disparidades entre os estados do Brasil quanto ao número de publicações.

## INTRODUCTION

The growing demand for structuring and organizing information gleaned from the scientific and academic domains has allowed healthcare scientists to analyze scientific output in unprecedented detail. Bibliometrics emerges as an important tool that enables one to map and generate different indicators of information management and knowledge.^1-5^

By implementing a bibliometric approach in Orthodontics one can evaluate publications that facilitate information retrieval and stratification. In view of the above, after reviewing, stratifying and electronically storing a catalog comprising the information identified, one can build an accessible database. This database can aid patients and professionals in making decisions about clinical procedures, as well as raise new research topics.^6^

After careful review of the orthodontic literature, Sun et al^6^ found clinically relevant information on which to base clinical decisions that struck a balance between etiology, diagnosis, treatment and prognosis. Mavropoulos and Kiliaridis^7^ found that articles published in orthodontic journals increasingly focused on treatment evaluation and diagnosis. They further found that the number of investigations into new treatment methods dwindled significantly over the past 20 years, which led them to conclude that there is absolute need for high-quality studies capable of generating reliable scientific evidence.

Nevertheless, few studies in Orthodontics make use of bibliometric analysis, evincing the need for further research carried out on the basis of this methodology.^7,8^ Therefore, the aim of this study is to describe the profiles of Brazilian and international publications in two journals of Orthodontics at three different intervals over a period of 10 years (1999 - 2004 - 2009), assessing the knowledge domains and article designs used, while identifying the country of origin of the published studies.

## MATERIAL AND METHODS

The journals used in this research were selected from the Brazilian National Council for Scientific and Technologic Development (CAPES) database. Only those Brazilian and international periodicals which ranked among the best in the area of Orthodontics according to CAPES-QUALIS rating in 2010 were selected. The selected journals were Dental Press Journal of Orthodontics (Dental Press Publishers, Maringá-PR, Brazil) and American Journal of Orthodontics and Dentofacial Orthopedics (Elsevier Publishers, Amsterdam, The Netherlands).

With the 1999, 2004 and 2009 issues of the selected journals in hand, copies of the abstracts of all articles were analyzed and divided by year and afterwards divided by volume and number. Subsequently, the titles and abstracts were reviewed.^9^ The following three variables were assessed: Knowledge domain, study design and countries where the article(s) were published. When it was not possible to characterize these research variables using only the title and the abstract, the full article was obtained and analyzed in the same manner. Two examiners were trained and calibrated to collect information. Their performance was measured by means of the Kappa test, of which result was k = 0.9654 (nearly perfect agreement). Categorization of data was carried out independently by physical, manual and direct review of the abstract of each reference. Preface, editorial, readers' letters and other data pertaining to journal organization were excluded.

Six knowledge domains and 18 specific subdomains were established in order to detect and assess trends in the themes and subjects being addressed. Study designs were ranked as advocated by Fletcher and Fletcher.^10^ The countries in the study were identified and named according to the country of origin of each author whose mailing address was disclosed, with the purpose of reaching the total number of publications in each country, thereby detailing their scientific contribution. When the author was Brazilian, their state of origin was also recorded according to their mailing address.

The instrument used to record the data included a specific form containing questions about each knowledge domain, study design and country of publication. The data were then organized and analyzed using Statistical Package for the Social Sciences (SPSS) software, version 10.0, presented by means of descriptive and analytical statistics.

To investigate whether there was a significant association between journals and countries of publication and the other study variables, the chi-square and Fisher's Exact tests were employed. These tests are used to verify the existence of a significant association between two qualitative variables. Fisher's exact test is an alternative to the chi-square test when samples are too small.

## RESULTS

The chi-square and Fisher's exact tests demonstrated that all variables (knowledge domain, study design and year) were significantly correlated with the journals analyzed. In terms of number of publications, there was a substantial difference between journals, indicating that such difference was maintained throughout the study period. This association proved statistically significant (p = 0.008) (Table 1).

**Table 1:** Distribution of articles for each journal evaluated..

Year	Journal				
	AJO-DO		Dental Press		p
	n	%	n	%	
1999	164	35.7	40	22.9	0.008^1^*
2004	134	29.1	63	36.0	
2009	162	35.2	72	41.1	
Total	460	100	175	100	

1 Chi-square test

* significant p ≤ 0.01.

The cohort, cross-sectional and *in vitro *laboratory studies proved the most frequent publications in the AJO-DO, whereas cross-sectional design, literature review and cohort studies were more frequently observed in the Dental Press Journal. The design variable was significantly associated with the journals (p = 0.000) (Table 2). The areas of Dental Material and Treatment Devices were the most frequently found in the AJO-DO. In the Dental Press Journal, on the other hand, Treatment Devices and Dental Malocclusion appeared to be the most frequent domains. Knowledge domain was also significantly associated with the Dental Press Journal (p = 0.000) (Table 2).

**Table 2:** Distribution of articles according to the variables evaluated.

Variable	Category	Journal				
		AJO-DO		Dental Press		P
		n	%	n	%	
Design	Case-control	1	0.2	-	-	0.000^2^*
	Cohort	110	23.9	30	17.1	
	Non-randomized clinical trial	13	2.8	3	1.7	
	Randomized clinical trial	28	6.1	1	0.6	
	*In vitro* laboratorial	87	18.9	16	9.1	
	*In vivo* laboratorial	33	7.2	3	1.7	
	Meta-analysis	2	0.4	-	-	
	Specialist's opinion	5	1.1	-	-	
	Case report	50	10.9	19	10.9	
	Systematic review	3	0.7	2	1.1	
	Literature review	20	4.3	43	24.6	
	Case series	8	1.7	8	4.6	
	Cross-sectional	100	21.7	50	28.6	
Knowledge domain	Skeletal anchorage	28	6.1	3	1.7	0.000^2^*
	Temporomandibular joint	25	5.4	5	2.9	
	Cariology	13	2.8	3	1.7	
	Surgery	32	7.0	14	8.0	
	Treatment devices	57	12.4	28	16.0	
	Pharmacology	8	1.7	-	-	
	Imaging	24	5.2	17	9.7	
	Dental material	78	17.0	15	8.6	
	Orthodontic mechanics	46	10.0	14	8.0	
	Morphology	48	10.4	18	10.3	
	Dental malocclusion	39	8.5	22	12.6	
	Legal Dentistry	-	-	5	2.9	
	Other	16	3.5	11	6.3	
	Special patients	2	0.4	-	-	
	Oral pathology	17	3.7	5	2.9	
	Follow—up	8	1.7	2	1.1	
	Bone and root resorption	8	1.7	8	4.6	
	Mouth breathing	1	0.2	3	1.7	
	Retention	9	2.0	2	1.1	
	Occlusal index	1	0.2	-	-	

2 Fisher's exact teste

* significant p ≤ 0.01

There were publications from 47 different countries. Both Brazilian and international journals predominantly published texts written by authors of their respective countries of origin. The United States was the country with the greatest number of published studies in the American Journal (162 articles, 35.2%), followed by Brazil (9.1%). Brazilian-authored studies prevailed in the Dental Press Journal, with 174 articles (99.4%) (Table 3).

**Table 3:** Countries of origin of published articles.

Country				Journal			
		AJO-DO				Dental Press	
	n		%		n		%
Brazil	42		9.1		174		99.4
USA	162		35.2		-		-
Japan	37		8.0		-		-
Turkey	24		5.2		-		-
England	19		4.1		-		-
Canada	18		3.9		-		-
Korea	18		3.9		-		-
Italy	17		3.7		-		-
Germany	14		3.0		-		-
Holland	10		2.2		-		-
Greece	8		1.7		-		-
Israel	8		1.7		-		-
India	8		1.7		-		-
China	7		1.5		-		-
Sweden	7		1.5		-		-
Switzerland	5		1.1		-		-
Taiwan	5		1.1		-		-
Austria	4		0.9		-		-
Australia	4		0.9		-		-
Belgium	3		0.7		-		-
Egypt	3		0.7		-		-
Kuwait	3		0.7		-		-
Mexico	3		0.7		-		-
Norway	3		0.7		-		-
Thailand	3		0.7		-		-
Spain	2		0.4		-		-
Finland	2		0.4		-		-
Ireland	2		0.4		-		-
Iceland	2		0.4		-		-
Others	17		3.7		1		0.6
Total	460		100		175		100

Brazilian publications comprised cross-sectional studies involving the study of malocclusion, imaging and morphology. Laboratory studies were generally correlated with research on dental material. Research on surgery and treatment devices are carried out by cohort studies (Table 4).

**Table 4:** Study design in relation to knowledge domain for the Brazilian journal.

Knowledge domain	Design									
	Cohort	Non-rand. clinical trial	Rand. clinical trial	I*n vitro* lab.	I*n vivo* lab.	Cross sect.	Case report	Literature review	Case series	Others
Skeletal anchorage	0	0	0	0	0	0	2	0	1	0
Temporomandibular joint	0	0	0	0	0	3	0	2	0	0
Cariology	1	1	1	0	0	0	0	0	0	0
Surgery	8	1	0	0	0	0	0	5	0	0
Treatment devices	9	1	0	1	0	2	7	6	2	0
Imaging	2	0	0	1	0	11	0	2	1	0
Dental malocclusion	1	0	0	0	0	11	5	2	2	1
Dental material	0	0	0	10	0	2	0	3	0	0
Orthodontic mechanics	3	0	0	1	2	1	2	5	0	0
Morphology	4	0	0	1	0	12	0	1	0	0
Retention	0	0	0	0	0	0	1	1	0	0
Oral pathology	1	0	0	0	1	0	0	1	1	1
Follow-up	1	0	0	0	0	0	0	1	0	0
Bone and root resorption	0	0	0	0	0	1	2	5	0	0
Others	0	0	0	2	0	7	0	9	1	0

In international publications, cohort research was most often associated with studies on treatment devices and morphology. *In vitro* laboratory studies, in turn, were found to be significantly linked to research on dental material. Research on morphology, dental malocclusion and imaging occurred most often in cross-sectional studies. In case reports, studies on dental malocclusion prevailed (Table 5).

**Table 5:** Study design in relation to knowledge domain for the international journal.

Knowledge domain	Design									
	Cohort	Non-rand. clinical trial	Rand. clinical trial	I*n vitro* lab.	I*n vivo* lab.	Cross sect.	Case report	Literature review	Case series	Others
Skeletal anchorage	5	0	1	3	5	6	5	0	1	2
Temporomandibular joint	7	0	0	2	4	9	0	0	2	1
Cariology	3	1	3	4	2	0	0	0	0	0
Surgery	13	1	2	2	3	1	9	1	0	0
Treatment devices	26	7	9	4	1	3	5	0	0	2
Pharmacology	0	1	2	0	4	0	0	0	0	1
Imaging	2	0	0	4	0	15	0	2	1	0
Dental malocclusion	3	0	2	0	0	20	12	2	0	0
Dental material	4	0	6	59	2	1	2	4	0	0
Orthodontic mechanics	10	2	2	6	8	5	8	1	1	3
Morphology	17	1	0	2	2	23	0	3	0	0
Retention	2	0	0	0	0	0	3	3	1	0
Oral pathology	7	0	0	1	0	4	2	1	2	0
Follow-up	7	0	0	0	0	0	1	0	0	0
Bone and root resorption	2	0	0	0	2	3	1	0	0	0
Others	2	0	1	0	0	10	2	3	0	2

São Paulo was the Brazilian state which produced the largest number of publications in both the Brazilian and the international journals. Paraná state ranked second in frequency of publications in the Brazilian journal. Minas Gerais state achieved the second highest frequency in the international journal and stood out, along with Rio de Janeiro state, in the Brazilian journal (Table 6).

**Table 6:** State of origin of Brazilian published articles.

State				Journal			
		AJO-DO				Dental Press	
	n		%		n		%
Outside Brazil	418		90.9		1		0.6
Alagoas	-		-		1		0.6
Bahia	-		-		4		2.3
Ceará	-		-		1		0.6
Distrito Federal	1		0.2		2		1.1
Espírito Santo	-		-		1		0.6
Goiás	-		-		2		1.1
Maranhão	-		-		2		1.1
Mato Grosso do Sul	-		-		2		1.1
Minas Gerais	7		1.5		15		8.6
Pará	-		-		2		1.1
Paraíba	-		-		2		1.1
Paraná	2		0.4		24		13.7
Pernambuco	1		0.2		2		1.1
Piauí	-		-		1		0.6
Rio de Janeiro	4		0.9		17		9.7
Rio Grande do Norte	-		-		3		1.7
Rio Grande do Sul	4		0.9		6		3.4
Santa Catarina	-		-		5		2.9
São Paulo	23		5.0		82		46.9
Total	460		100.0		175		100.0

## DISCUSSION

Contemporary Orthodontics has shown a considerable interest in themes that contribute to strengthening the interaction between scientific evidence and clinical decisions. As a result, the quality of studies available in the literature is an important factor which requires investigation. This study performed a bibliometric analysis of orthodontic literature with the purpose of characterizing scientific output in this research area. Brazilian and international abstracts of high-ranking scientific journals were used. Journals constitute not only an important communication channel for science, but also the most extremely dynamic method employed to disseminate knowledge developed in this dental specialty domain. These periodicals also convey the scientific output of a specific area. Furthermore, they boast utmost credibility and widespread dissemination among dental professionals.

Setting criteria to assess the influence of each scientific journal has aroused fruitful debates. The influence of a given journal can be determined in various ways such as by citation index, measured by impact factor (IF) or through its wide acceptance as indicated by how extensively it circulates. Kanavakis et al^8^ adopted the impact factor index as an inclusion criterion in their orthodontic bibliometric investigation. This analysis used CAPES-QUALIS as its frame of reference, since no Brazilian orthodontic journal currently has an impact factor.

In this study, as in the study conducted by Poletto and Faraco Júnior,^11^ all data were collected by exploratory reading of the title and abstract carried out by two specialists in Orthodontics, who worked separately and concurrently after training and calibration in order to impart reliability to the assessment and avoid measurement bias, as described in Nainar's study.^12^

Analysis of the articles comprised the years 1999, 2004 and 2009, spanning a period of 10 years, totaling 635 articles. Throughout the study period, the Brazilian journal published an increasing number of articles while the international journal remained superior and constant in the number of publications. It is worth noting that the Brazilian journal is a bimonthly publication of which inception occurred 16 years ago. The international journal, in turn, is a monthly publication launched 97 years ago. In the study carried out by Gibson et al,^13^ all articles published between 1999 and 2008 were collected, 425 of which were randomly analyzed so as to determine the publishing profile of four international orthodontic journals. With a view to assessing the methodology and quality of systematic reviews in Orthodontics, Papageorgiou et al^14^ resorted to electronic searches. In this research, however, collecting all articles published at 3 different intervals made it possible to compare trends in these publications over the years.

Harrison et al.^15^ found significant differences in the content of international journals of Orthodontics, suggesting that journals can depict different aspects of the specialty. An insight into the publishing trends of various journals can help clinicians to identify journals that are best suited to meet their needs.

Out of all research conducted by Gibson and Harrison,^13^ articles on growth and development, diagnosis and treatment accounted for 72.2% of the articles analyzed. This research showed similarity between the Brazilian and the international journal in terms of the domains studied, since the results indicate a trend towards the performance of studies on treatment devices and morphology in both journals. When classifying the knowledge domains in this investigation, under the "treatment devices" variable, for example, studies which aimed at assessing the efficacy and therapeutic characteristics of various appliances used in orthodontic treatment were included. In addition, studies on facial and bone analysis, as well as growth characteristics, among others, were included under morphology, in the respective knowledge domain. Thus, it was noted a trend towards interventional studies, suggesting interest in therapeutic clinical resolutions and their respective responses in the maxillofacial complex. The international journal published studies on dental material (17%) with a higher frequency. These studies evaluated, for example, mechanical and adhesive properties, or rate of poisoning of the material involved in the treatment. A plausible explanation for this percentage is the modernization of material, development of new products and methods of evaluation.

Epidemiological studies on malocclusion - dental abnormalities - were classified in the knowledge domain under "dental malocclusion," and had the second highest frequency rate (12.6%) in the Brazilian journal. These studies enable the implementation of preventive measures based on epidemiological surveys on the prevalence of malocclusion, which also provide an important foundation to assess the current condition and future oral healthcare needs of a population.

The cohort (23.9%) and cross-sectional (21.7%) study designs were the most frequent in the international journal. In the Brazilian journal, however, cross-sectional studies showed a 28.6% frequency, with literature reviews ranking second with 24.6%.

Laboratory studies are usually an important step that allow new treatment devices or material to be tested in animals to detect potential complications and unexpected results. These studies were highlighted in the international journals, showing a frequency rate of 26.1% when incorporated to *in vitro *and *in vivo* laboratory studies. Although they are necessary to establish basic quality parameters prior to application of material and techniques in humans, *in vitro* studies do not set guidelines for clinical conduct, nor do they afford critical information for clinical decision-making.^16^

Cross-sectional studies are a useful tool for describing characteristics of a population, identifying at-risk groups, for healthcare measures and planning, in addition to enabling research on the potential causal relationships between factors suspected of entailing risk or disease.^17^ In the present study, cross-sectional studies prevailed in the Brazilian journal (28.6% vs. 21.7% in the international journal). In Brazilian and international publications, cross-sectional studies were most frequently performed in research conducted on imaging, dental malocclusion and morphology.

Cohort studies are designed to investigate changes over time, i.e., to monitor a sequence of events.^18^ In the publications analyzed in this study, cohort research was often related to studies on treatment devices and morphology in the international journal, whereas it was related to surgery in the Brazilian publication.

Case reports and case series play a significant part in the scientific literature, as noted in this study. In their study, Kanavakis et al^8^ reviewed three international journals and found that case reports accounted for 8.89% of the designs evaluated (AJO-DO only: 11.06%). These results resemble those found in this study, if case reports and case series are grouped together (12.6% in AJO-DO, and 15.5% in the Dental Press Journal).

Randomized clinical trials provide the best evidence quality available, and, therefore, it is extremely important to assess how they are conducted and reported. The results of randomized clinical trials influence decision-making in clinical practice and constitute the backbone of systematic reviews.^14,19^ A study conducted by Shimada et al^20^ identified 161 randomized clinical trials in orthodontic journals issued between 2003 and 2007. This study showed that in both journals there were only a few clinical trials. Additionally, one can expect the number of well conducted trials to be even lower, considering design criteria such as actual randomization of the trial groups, presence of a control group, blinding and standardization of criteria. Thus, the data are in line with research by Pandis et al^19^ who found that the quality of randomized clinical trials in the main Dentistry journals can be considered low, and warned that study design is of paramount importance to improve dental treatment. Gibson and Harrison,^13^ however, estimate that the number of randomized clinical trials in orthodontic journals has risen dramatically in recent years.

In this study, systematic reviews and meta-analyses accounted for less than 1% of all reviewed articles, underscoring the need for expanding knowledge of evidence-based methods among researchers of Orthodontics as well as in the general area of Dentistry. Defined by the literature as of moderate quality, no significant improvement was observed in these designs over the last decade.^14^

Literature reviews (24.6%) ranked second in the studies published in the Brazilian journal. This type of article can provide summaries of a particular subject, including relevant, updated information. Authors, however, usually employ informal and subjective methods when collecting and interpreting the studies, which could lead them to expose their own biases. In the hierarchy of studies, these are classified as low-power studies as they provide little evidence.

To determine article provenance, the methodology used by Yang and Zhao^21^ was applied and publications from 47 different countries were recorded. Most Brazilian and international journals predominantly published texts written by authors of their respective countries of origin. U.S. authors had the largest number of articles published in the American Dental Journal, with 162 (35.2%), while most studies published in the Dental Press Journal were written by Brazilian authors, i.e., 174 articles (99.4%).

Remarkably, it was found that journals tend to publish articles authored in their country of origin. Among the possible explanations for this phenomenon - and this seems to be the case of the Dental Press Journal - are the language in which the journal publishes the articles, and the databases used to index it. In 2010, the Brazilian journal began to be officially published in the English language as well. Nevertheless, it continues to be published in Brazilian Portuguese, in both print and electronic media. This change aimed at meeting the requirements of evaluation and indexation agencies in terms of visibility, impact factor and citation index of the journal.

According to Kanavakis et al,^8^ articles hailing from the U.S. made the bulk of AJO-DO publications. Moreover, the researchers reported that most texts published in the European Journal of Orthodontics (EJO) originated from Europe, further illustrating the trend towards publishing articles authored in the country of origin of the journal.

Brazil ranked second in the number of articles published in the AJO-DO. Dental research in Brazil has made major strides in recent years, mainly buoyed by the widespread circulation of specific-area journals and scientific papers presented at scientific meetings.^22,23^ According to Suehiro et al,^24^ this fact is basically due to an increase in the number of theses and dissertations published, the proliferation of undergraduate and graduate programs, a greater diversity of themes, innovation and methodological resources as well as strong social commitment, among others.

São Paulo state produced the largest number of publications in both Brazilian and international journals. Paraná state ranked second in frequency of Brazilian publications. Minas Gerais achieved the second highest frequency in the international journal and stood out, along with Rio de Janeiro state, in the Brazilian journal (Table 6). São Paulo boasts a larger number of universities and graduate programs in Dentistry, in addition to being the state with the largest GDP in Brazil, fostering research and the submission of an increasingly significant number of articles for publication.

The search for data in journals, which can be used to support professional decision-making, should be an ongoing process, based on the principles of evidence-based practice. Therefore, evaluating and employing articles that may provide grounds for informed clinical decision-making is part of continuing education for dental students and professionals alike. It should be emphasized that, ultimately, deploying strategies such as these can improve the efficiency of readers who aim at incorporating research results in their daily practice.

## CONCLUSIONS

Based on the methods used in this study, it is reasonable to conclude that most published articles involve studies with a low potential to generate evidence-based, scientific knowledge, thereby demonstrating that further research - grounded in better quality designs - is warranted. Furthermore, the literature reflects ongoing trends in clinical orthodontic practice, and discloses a relative similarity between Brazilian and international journals regarding the publication of studies on dental material and devices/appliances used in clinical practice. The need for laying down criteria regulating scientific output and publication in Brazilian graduate Dentistry programs, aided by the quality of Brazilian Orthodontics, can help to shed further light on the data presented in this article.
